# Objective Assessment of Acute Pain in Foals Using a Facial Expression-Based Pain Scale

**DOI:** 10.3390/ani10091610

**Published:** 2020-09-10

**Authors:** Johannes van Loon, Nicole Verhaar, Els van den Berg, Sarah Ross, Janny de Grauw

**Affiliations:** 1 Department of Clinical Sciences, Faculty of Veterinary Medicine, Utrecht University, 3584CM Utrecht, The Netherlands; J.C.deGrauw@uu.nl; 2 Clinic for Horses, University of Veterinary Medicine Hannover, 30559 Hannover, Germany; Nicole.Verhaar@tiho-hannover.de; 3 Dierenhospitaal Visdonk, 4707PE Roosendaal, The Netherlands; emhvandenberg@gmail.com; 4 Anglesey Lodge Hospital, The Curragh, Kildare R56 YX98, Ireland; sarahross60@hotmail.com

**Keywords:** pain, foal, facial expression, behaviour, equine, analgesia

## Abstract

**Simple Summary:**

Research has shown that objective assessment of pain in horses can be performed by subjectively scoring facial expressions. So far, no studies have been conducted to develop a pain measuring tool for the assessment of pain in foals. In other species like pigs and sheep, facial expressions have been shown to be good indicators of pain in neonatal animals. In this study, a pain scale that is already available for mature horses (EQUUS-FAP: Equine Utrecht University Scale for Facial Assessment of Pain) was adapted to measure different types of acute pain in neonatal and older foals with acute pain based on facial expressions. The scale was based on a pain scale that has been shown to be useful in mature horses with various types of acute pain (colic, orthopaedic, and head-related pain). This pain scale was tested in 20 patients with different types of acute pain (colic, laminitis, postoperative pain) and 39 healthy control animals. The authors found that the EQUUS-FAP FOAL (Equine Utrecht University Scale for Facial Assessment of Pain in Foals) is a reproducible pain scale that can be used to assess pain in neonatal and older foals.

**Abstract:**

Pain assessment is very important for monitoring welfare and quality of life in horses. To date, no studies have described pain scales for objective assessment of pain in foals. Studies in other species have shown that facial expression can be used in neonatal animals for objective assessment of acute pain. The aim of the current study was to adapt a facial expression-based pain scale for assessment of acute pain in mature horses for valid pain assessment in foals. The scale was applied to fifty-nine foals (20 patients and 39 healthy controls); animals were assessed from video recordings (30–60 s) by 3 observers, who were blinded for the condition of the animals. Patients were diagnosed with acute health problems by means of clinical examination and additional diagnostic procedures. EQUUS-FAP FOAL (Equine Utrecht University Scale for Facial Assessment of Pain in Foals) showed good inter- and intra-observer reliability (Cronbach’s alpha = 0.95 and 0.98, *p* < 0.001). Patients had significantly higher pain scores compared to controls (*p* < 0.001) and the pain scores decreased after treatment with NSAIDs (meloxicam or flunixin meglumine IV) (*p* < 0.05). Our results indicate that a facial expression-based pain scale could be useful for the assessment of acute pain in foals. Further studies are needed to validate this pain scale.

## 1. Introduction

Objective pain assessment in horses has received much attention in the last decades [1,2] and facial-expression-based pain scales have been described for horses [3,4], mice [5], and cats [6]. The EQUUS-FAP (Equine Utrecht University Scale for Facial Assessment of Pain) is a facial expression-based pain scale that has been described in horses with acute colic pain [7], acute head-related pain [8], and acute and postoperative orthopaedic pain [9]. However, no studies have described pain scales for objective assessment of pain in foals.

Robertson [10] described the changes in physiological parameters from birth to adulthood in horses and the importance of behavioural expressions in recognizing pain in neonatal foals. Although Robertson [10] stressed the importance of validated pain scoring systems in foals, no such scales have been developed and tested in foals to date. O’Neill et al. [11] examined the developmental trajectories of infant facial expressions of pain during the first year of life. They described changes in distress regulation influencing pain expression. Infants showed decreasing signs of intense distress and sensory overload (shown by tightly shutting the eyes with a cry mouth) when aging from 2 months of age to 6 months of age.

Studies in other species have shown that facial expression can be used in neonatal animals for objective assessment of pain, like the piglet and lamb grimace scale [12,13]. Vullo et al. [14] found that the Piglet Grimace Scale proved to be effective to assess pain associated with cryptorchidectomy in neonatal piglets. Further studies are needed to validate the Piglet Grimace Scale for other potentially painful procedures, but this study showed that facial expressions could potentially be used in neonatal mammals to assess pain. Since foals experience potentially painful conditions like acute colic, trauma, orthopaedic pain, and need surgery for certain diseases, leading to postoperative pain, there is a clear need for objective pain assessment in foals as well. Pharmacokinetic and pharmacodynamic studies on analgesic drugs in foals are broadly missing and validated pain scales for foals could help define accurate and effective dosing regimens in foals. 

The aim of the current study was to develop a facial expression-based pain scale for the assessment of acute pain in neonatal and older foals (EQUUS-FAP FOAL). The hypothesis was that EQUUS-FAP FOAL would be a valid and repeatable pain scale for the assessment of acute pain in foals. Validity and clinical applicability were investigated by applying this pain scale to a cohort of patients with several types of acute pain and healthy pain-free control foals. 

## 2. Materials and Methods 

Fifty-nine foals (20 patients and 39 healthy control animals), assessed at different locations, were used in this study. Attending veterinarians from different clinics (in Germany, Ireland, and the Netherlands) selected patients that were diagnosed with health problems leading to acute pain (due to trauma, laminitis, gastic ulcers, orthopaedic or visceral pain, and postoperative pain) and that were accompanied by their dam. Foals that were without their mare or that had altered mental status (like Perinatal Asphyxia Syndrome or liver disease, for instance) were excluded from the study. Besides the patient group, a control group was selected comprising clinically normal healthy foals. Both patients and control foals were divided into a neonatal group (0–14 days of age) and a group of older foals (15 days to 6 months of age) (Table 1). These healthy control foals were partly assessed in the different clinics that were involved in this study and also assessed at some breeding farms. More details of the patients can be found in Supplementary Materials Table S1. Written informed consent was obtained from all owners. Video recordings of the subjects were obtained with the foals loose and unrestrained in a stable with their dam in order to minimize the potential for stress and/or distraction to influence facial expression. Video clips ranged in duration from 30 to 60 s because foals sometimes hide behind their mare. The time that the foals were visible for the observers was standardized as much as possible to 30 s. The videos were taken by a person outside of the box by means of a smartphone camera. The persons acquiring the videos were treating veterinarians or students. Patients were filmed at different time points, directly before and between 1–2 h after analgesic treatment with NSAIDs (being either flunixin meglumine 1.1 mg/kg IV or meloxicam 0.6 mg/kg IV). Videos were blinded and randomized. Video observations were performed by three observers (1 senior anaesthetist and 2 veterinary master students) who were blinded for the condition and the analgesic treatment regimens of the patients, using the EQUUS-FAP FOAL pain scale. The students had brief training (over 2 days) by their supervisor (the senior anaesthetist) by means of various photographs and video recordings of mature horses and foals that were not included in the study. The students observed all videos twice with an interval of 2 weeks in a randomized order to assess intra-observer agreement. EQUUS-FAP FOAL (with a range of pain scores from zero to 22) is based on the EQUUS-FAP for mature horses, with some slight adaptations (Table 2). Firstly, the visibility of the sclera was omitted from the parameter “eyelids” because pilot observations revealed that many healthy pain-free foals also show their sclera. Furthermore, the facial action unit “flehmen” was removed, because many foals displayed this behaviour during pain-free conditions as well. Pilot observations in foals with acute pain showed lip-smacking; therefore, we added this facial action unit to the pain score. Yawning, teeth grinding, and moaning were all included as individual facial action units in the pain score. These changes were developed by the senior anaesthetist involved in the study. The new ethogram has been tested in 15 foals (that were not included in this study) before the start of the study (unpublished results).

The pain scores were assigned by means of a score table filled out during video observations. The age of patients and control foals was compared by means of independent samples T-tests, and the distribution of sex for the patients and control foals was assessed by means of Pearson Chi-square tests (for neonatal and older foals separately). All pain scoring data are expressed as medians and quartiles. Inter- and intra-observer agreement for total pain scores were assessed by means of Intra Class Correlation (ICC) analysis. Differences between neonatal and older control foals and between patients and controls were tested using Mann Whitney U tests. Differences in pain scores before and 1–2 h after NSAID-treatment in twelve patients were tested with the Wilcoxon signed rank test. Cut-off values for the pain scales to differentiate between patients and control animals were determined by means of ROC (Receiver Operating Characteristic) analysis and sensitivity and specificity were calculated. Statistical analysis was performed using commercially available software (SPSS version 20.0, IBM). Statistical significance was accepted at *p* < 0.05. 

## 3. Results

**Figure 1:**
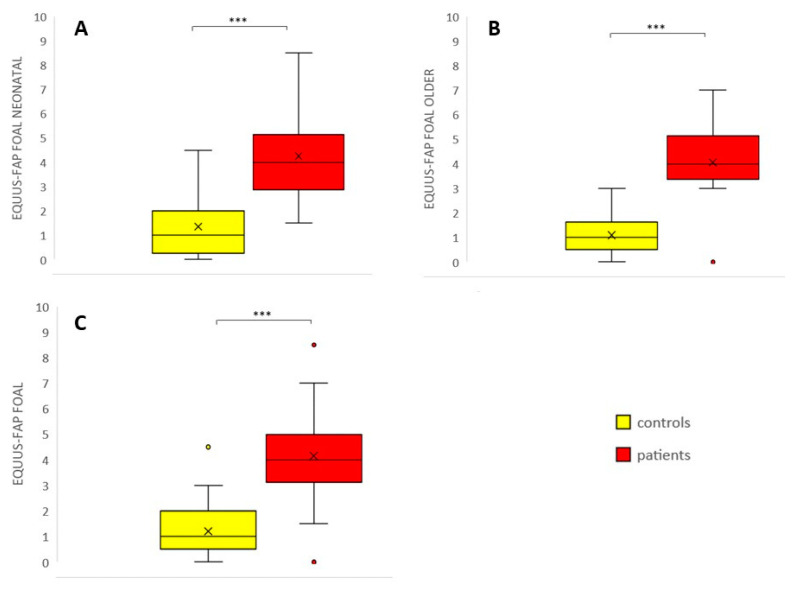
EQUUS-FAP FOAL (Equine Utrecht University Scale for Facial Pain Assessment for foals) patients versus controls; (**A**): EQUUS-FAP FOAL in neonatal foals (0–14 days) (*n* = 10 patients, *n* = 17 controls); (**B**) EQUUS-FAP FOAL in older foals (14 days–6 months) (*n* = 10 patients, *n* = 22 controls); (**C**): EQUUS-FAP FOAL in all foals (*n* = 20 patients, *n* = 39 controls). Lines in boxes show median scores; boxes show 25–75th percentiles; error bars show 5–95th percentiles and outliers are shown as dots. *** = *p* < 0.001.

**Figure 2:**
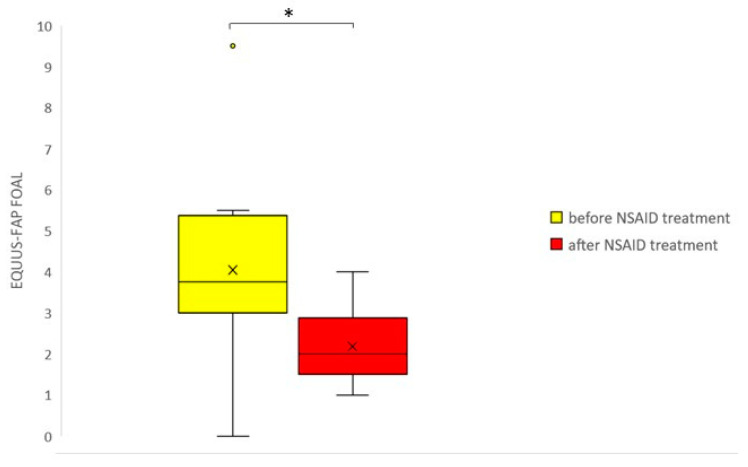
EQUUS-FAP FOAL (Equine Utrecht University Scale for Facial Pain Assessment for foals) in patients, directly before versus 1–2 h after NSAID (Non-Steroidal Anti Inflammatory Drugs) treatment (either Flunixin meglumine or Meloxicam IV). Lines in boxes show median scores; boxes show 25–75th percentiles; error bars show 5–95th percentiles and outliers are shown as dots.* = *p* < 0.05 (*n* = 12).

**Figure 3:**
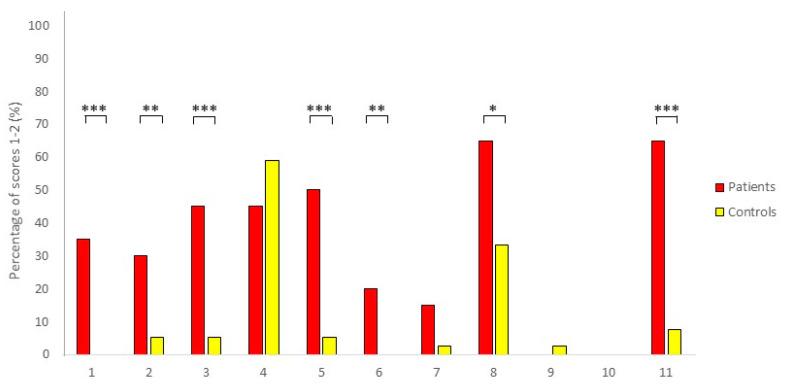
EQUUS-FAP FOAL (Equine Utrecht University Scale for Facial Pain Assessment for foals) frequency distribution; facial action unit 1: head movement, facial action unit 2: eyelids, facial action unit 3: focus, facial action unit 4: nostrils, facial action unit 5: corners mouth/lips, facial action unit 6: muscle tone, facial action unit 7: yawning, facial action unit 8: smacking with the lips, facial action unit 9: teeth grinding, facial action unit 10: moaning, facial action unit 11: ears. Percentage of scores 1–2 (%) = percentage of foals that had a score of 1 or above for each facial action unit, * = *p* < 0.05, ** = *p* < 0.01, *** = *p* < 0.001. (*n* = 20 patients, *n* = 39 controls).

**Figure 4:**
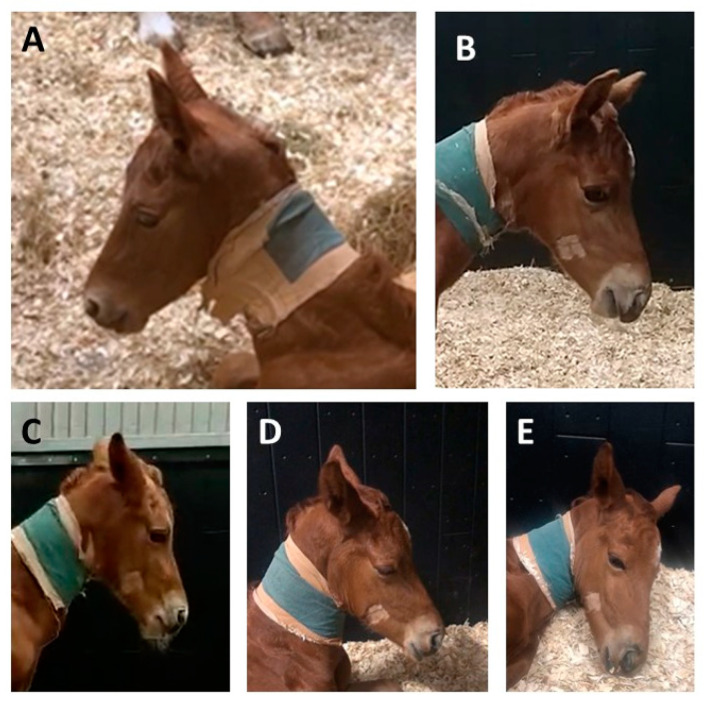
(**A**–**E**) Examples of 9 days old Warmblood foal after surgery for proximal phalanx fracture.

Neonatal patient foals had a significantly higher age compared to the neonatal control foals (*p* < 0.01); for the older foals, patients and control foals did not have significantly different ages (*p* = 0.5). The distribution of sex was not statistically different between patients and controls in the neonatal (*p* = 0.35) and older foals (*p* = 0.08). Table 3 shows the results of correlation analysis between the total pain scores of three independent observers. Inter-observer agreement was strong and significant for all foals together (Cronbach’s alpha = 0.95, *p* < 0.001 over 3 observers); intra-observer agreement was strong and significant as well (Cronbach´s alpha = 0.98, *p* < 0.001 over 2 observers). Inter-observer agreement was higher for neonatal foals compared to older foals (0.97 versus 0.90 respectively, Table 3). Neonatal and older control foals did not show differences in pain scores (*p* = 0.61). Patients had significantly higher pain scores compared to control animals, both for neonatal (0–14 days) and older foals (14 days–6 months) and for the total group (*p* < 0.001 for all groups) (Figure 1). Pain scores significantly decreased following the administration of NSAIDs (*p* < 0.05) (Figure 2). Using a cut-off value of >3 to discriminate between patients and healthy control foals, the sensitivity of EQUUS-FAP FOAL was 90%, and specificity was 84.6%. Figure 3 shows the frequency distribution of positive scores (1 or 2) of all individual facial action units of EQUUS-FAP FOAL. Statistical differences in positive scores between patients and controls were found for the following individual facial action units: head, eyelids, focus, corners of the mouth, muscle tone, smacking of the lips, and ears. More details of the individual EQUUS-FAP foal scores for both neonatal and older foals can be found in Supplementary Materials Tables S2–S7. Figure 4 shows photographs of a 9-day-old colt Warmblood foal that was admitted with a proximal phalanx fracture that was treated surgically. This figure shows specific elements of the EQUUS-FAP FOAL. 

Panel A shows a bright and alert foal 2 h after NSAID treatment on day 2 after surgery. The position of the eyelids cannot be completely evaluated due to the direction of the camera. Panel B shows strained corners of the mouth (score 2) and a slightly raised upper eyelid (score 1). Panel C shows backwards-directed ears (score 2) and slightly strained corners of the mouth (score 1). Panel D shows the backwards position of the ears (score 2) and tightened eyelids (score 2), while the corners of the mouth are relaxed (score 0). Panel E shows the foal during rest, with slightly tightened eyelids (score 1) and the ears directed to the sides (score 1). Panels B–E were all acquired during the first day after surgery, in the afternoon after IV Flunixin Meglumine administration in the morning (between 3 and 6 h after administration). Total EQUUS-FAP FOAL pain score for day 2 after surgery (Panel A) was 1, Total EQUUS-FAP FOAL pain score for day 1 after surgery (Panels B–E) varied between 3 and 8.

## 4. Discussion

The current study shows that the EQUUS-FAP FOAL pain scale that is based on facial expressions in neonatal and older foals proved to be valid and clinically applicable for assessment of acute pain in these foals. The hypothesis was therefore accepted; the scale showed good intra- and inter-observer agreement, with a higher agreement between the observers for the neonatal foals compared to the older foals. EQUUS-FAP FOAL differentiated between patients and controls and a decrease of pain scores was found after NSAID treatment in patients suffering from acute pain. 

The most discriminative facial action units of the EQUUS-FAP FOAL for neonatal and older foals were head movement, the position of the eyelids (closed eyelids or raised upper eyelid), focus, corners of the mouth, lip-smacking, and position of the ears. The EQUUS-FAP FOAL was based on the EQUUS-FAP pain scale for mature horses, with some small adaptations. First, the visibility of the sclera was omitted from the facial action unit “eyelids” because many healthy pain-free foals also show their sclera. Furthermore, the facial action unit “flehmen” was removed, because many foals displayed “flehmen” during pain-free conditions as well. Although “flehmen” has been described as possible pain behaviour in, for instance, horses with colic pain [7], the flehmen response acts in conjunction with the vomeronasal organ near the palate to amplify smells. As we saw this behaviour multiple times in healthy foals, it may play a more important role in discovering new sensations. Flehmen has been described in foals that are free of pain [15], with higher frequencies in healthy colts, starting already at 2 days of age and decreasing in frequency over the first 4 weeks of age. In fillies, flehmen frequency was lower but did not decrease over time. In a more recent study [16], this pattern of flehmen in young foals was again investigated, and flehmen was seen in both colts and fillies that were not in pain. Since we observed several foals with acute pain displaying periods of smacking of the lips, we added this behaviour to the pain score. Apart from that, we separated the facial action units yawning, teeth grinding, and moaning in order to be better able to assess the importance of individual parameters afterwards. Increased muscle tone, moaning, yawning and teeth grinding were the least discriminative in our scale, since they were rarely seen in patients and control foals. The nostril facial action unit was scored positive very often both in patients and control foals and was therefore also not discriminative. We speculate that the higher breathing frequency that foals display also influences the conformation and opening of their nostrils, making this facial action unit not suitable to indicate any painful condition in foals. 

Our findings in foals are in line with a study in lambs [13] that described similar changes in facial expressions after tail-docking. The Lamb Grimace Scale consists of five facial action units: orbital tightening, mouth features, nose features, cheek flattening, and ear posture. These authors found mouth features (flattened and tightened lips) and orbital tightening showing significant quantitative changes after tail docking. Both types of changes are also seen in foals with acute pain. Vullo et al. [14] described a piglet grimace scale that is based on 3 facial action units: ear position, orbital tightening, and cheek tightening/nose bulge. The pain scores increased 6 h after cryptorchidectomy surgery, and piglets showed less activity and lying down with pen mates. Ear position and orbital tightening were also identified as important facial action units in our foals suffering from acute pain. Both the Lamb Grimace Scale and the Piglet Grimace Scale are limited to fewer facial action units compared to the current study for foals, but the similarities in facial expressions seen with acute pain for the different species are obvious, and in both lambs and piglets facial expressions could be used to assess acute pain as well. 

We found slightly higher agreement between the observers for the neonatal foals compared to the older foals. This difference in agreement could possibly be explained by the fact that older foals were more exploratory and reactive to the environment, making it slightly more difficult to assess facial expressions. This exploratory behaviour is something we observed in the older foals more often. Specifically, inter-observer agreement was lower for older foals, although still acceptable. For intra-observer agreement, the difference between neonatal and older foals was much smaller, with slightly better agreement for the neonatal foals. Considering the relatively difficult task of assessing various facial action units from a very short video assessed at real-time normal speed, the agreement that was reported in the current study is rated as good to excellent. The Master’s students that were participating in this study were trained to observe the videos briefly before the study. For training purposes, both videos and live observations of mature horses were used, and later, videos of neonatal foals that were not involved in this study were used and discussed after training.

A limitation of the current study was that the number of patients included was relatively low, making it impossible to compare different types of pain and, for instance, the influence of breed and gender on the expression of pain. In a follow-up study, new patients will be enrolled in order to compare different types of pain to a further extent. Furthermore, differences between foals from different breeds or gender will be assessed also. Another limitation of the current study was that, due to clinical circumstances, the blinding of the condition of the foals with acute pain was sometimes not completely possible. In order to prevent the influence of this possible bias, we have included healthy pain-free foals that were housed under clinical circumstances as well. Furthermore, foals with acute pain were also assessed before and after the administration of analgesic drugs. The observers were unaware of the treatment of the foals and they were also unaware of the day after surgery or onset of acute pain when assessing videos. It is true that in some foals, the visibility of a neck bandage or a clipped area showed that this foal had treatment or surgery, but this was not the case for all foals that were included in this study with acute pain. In this way, we have tried to prevent bias as much as possible while sticking to the clinical conditions that had to be accepted in the current set-up of the study. Due to the relatively low numbers of patients, we did not perform any statistical analyses on possible differences between facial expressions of foals with different types of acute pain. However, these individual foals with different types of acute pain did not appear to show differences in their facial expressions. Earlier studies in mature horses [3,4,8,9] have shown that horses suffering from different types of acute pain show clear similarities in their facial expressions. In follow-up studies with larger groups of patients, we aim to analyze possible differences in facial expressions in foals with different types of pain.

## 5. Conclusions

This study suggests that it is possible to use a facial expression-based pain scale for assessment of acute pain in foals. Inter- and intra-observer agreement were good to excellent and it was possible to discriminate foals with acute pain from healthy pain-free foals. Further studies are needed to further validate this pain scale before it can be used in clinical patients.

## Figures and Tables

**Table 1:** Data of foals that were included in the study (*n* = 59).

Patient Details	Patients	Controls
Number of neonatal foals	10	17
Mean age in days (SD) neonatal foals	10.6 (4.4) *	5.4 (2.6)
Number of colts	6	7
Number of fillies	4	10
Number of older foals	10	22
Mean age in days (SD) older foals	102.9 (39.1)	112.2 (39.8)
Number of colts	1	9
Number of fillies	9	13
Total number of foals	20	39

Neonatal foals are between 0–14 days, older foals are between 15–180 days. SD = standard deviation, * = *p* < 0.01.

**Table 2:** Score sheet of the Equine Utrecht University Scale for Facial Pain Assessment for foals (EQUUS-FAP FOAL).

Facial Action Units	Categories	Score
Head	Normal head movement	0
	Less movement/increased movement	1
	No movement/strongly increased movement	2
Eyelids	Opened, sclera can be seen in case of eye/head movement	0
	More opened eyes/tightening of eyelids	1
	Obviously more opened eyes/obvious tightening of eyelids	2
Focus	Focused on environment	0
	Less focused on environment	1
	Not focused on environment	2
Nostrils	Relaxed	0
	A bit more opened	1
	Obviously more opened, nostril flaring and possibly audible breathing	2
Corners mouth/lips	Relaxed	0
	Lifted slightly	1
	Obviously lifted	2
Muscle tone head	No fasciculations	0
	Mild fasciculations	1
	Obvious fasciculations	2
Yawning	Not seen	0
	Seen	2
Smacking with the lips	Not seen	0
	Seen	2
Teeth grinding	Not heard	0
	Heard	2
Moaning	Not heard	0
	Heard	2
Ears	Position: orientation towards sound/clear response with both ears or ear closest to source	0
	Delayed/reduced response to sounds	1
	Position: backwards/no response to sounds	2
Total score		/22

**Table 3:** Inter- and intra-observer agreement of total pain scores of EQUUS-FAP FOAL (Equine Utrecht University Scale for Facial Pain Assessment for foals).

Agreement	Neonatal Foals	Older Foals
Inter-observer agreement		
ICC observer 1–2	0.91	0.7
ICC observer 1–3	0.91	0.7
ICC observer 2–3	0.92	0.86
Crohnbach’s alpha	0.97	0.9
95% confidence interval	0.97–0.99	0.79–0.93
*p*-value	<0.001	<0.001
Intra-observer agreement		
Crohnbach’s alpha	0.98	0.96
95% confidence interval	0.97–0.99	0.94–0.97
*p*-value	<0.001	<0.001

ICC = Intra Class Correlation coefficient. For neonatal foals (0–14 days), *n* = 32 for inter-observer and intra-observer agreement. For older foals (15 days–6 months), *n* = 48 for inter-observer and intra-observer agreement.

## Supplementary Materials

The following files are available online at https://www.mdpi.com/2076-2615/10/9/1610/s1, Table S1: Details on patients and healthy control foals, Table S2: EQUUS-FAP FOAL scores of observer 1 for the neonatal foals, Table S3: EQUUS-FAP FOAL scores of observer 2 for the neonatal foals, Table S4: EQUUS-FAP FOAL scores of observer 3 for the neonatal foals, Table S5: EQUUS-FAP FOAL scores of observer 1 for the older foals, Table S6: EQUUS-FAP FOAL scores of observer 2 for the older foals, Table S7: EQUUS-FAP FOAL scores of observer 3 for the older foals.
