# Supplementary material for: Objective Assessment of Acute Pain in Foals Using a Facial Expression-Based Pain Scale
Source: Animals (Basel). 2020 Sep 10;10(9):1610. doi: 10.3390/ani10091610 (PMC7552134; doi:10.3390/ani10091610)
Supplement: Supplementary file 1 [file animals-10-01610-s001.zip › supplementary material 7 pain scores older foals Observer 3.pdf]

video number	head	eyelids	focus	nostrils	corners mouth/li	muscle tone head
1	0	0	0	1	0	0
2	0	1	0	0	1	0
4	0	1	0	0	1	0
5	0	0	0	0	0	0
6	1	1	0	0	0	0
7	0	0	0	1	1	0
8	0	1	0	0	0	0
10	0	1	0	0	0	0
11	0	0	0	1	1	0
12	0	1	0	1	0	0
13	0	0	0	1	0	0
14	0	1	0	1	1	0
15	0	1	0	0	0	0
16	0	1	0	1	1	0
17	1	1	1	0	1	0
18	0	1	0	0	1	0
19	0	1	0	0	0	0
20	0	0	0	0	0	0
21	0	1	0	1	1	0
22	0	1	0	0	0	0
23	0	1	0	0	0	0
24	0	1	0	0	0	0
25	0	1	0	0	1	0
26	0	1	0	1	1	0
27	0	1	0	1	0	0
28	0	1	0	1	1	0
30	1	1	0	1	0	0
31	0	0	0	1	0	0
32	0	0	0	1	1	0
33	0	1	1	1	1	0
34	0	1	0	1	1	0
35	0	1	0	0	0	0
36	0	1	0	1	1	0
37	0	0	0	1	1	0
38	0	1	0	1	0	0
39	0	0	0	1	0	0
40	0	1	0	0	1	0
41	0	1	0	0	1	0
42	0	0	0	1	0	0
43	0	1	0	1	0	0
44	0	0	0	1	0	0
45	0	1	0	1	1	0
46	0	1	0	0	0	0
47	0	0	0	1	0	0
48	0	1	1	0	0	0
49	0	1	0	0	1	0
50	0	0	0	1	0	0
51	0	1	0	1	1	0
52	0	1	0	0	0	0

53	0	1	1	1	0	0
55	0	0	0	1	0	0

video number

1	0	0	0	0	0	1
2	0	0	0	0	0	2
4	0	2	0	0	1	5
5	0	0	0	0	0	0
6	0	0	0	0	0	2
7	0	2	0	0	0	4
8	0	0	0	0	0	1
10	0	0	0	0	0	1
11	0	2	0	0	0	4
12	0	0	0	0	0	2
13	0	0	0	0	0	1
14	0	0	0	0	0	3
15	0	0	0	0	1	2
16	0	0	0	0	0	3
17	0	0	0	0	1	5
18	0	2	0	0	0	4
19	0	0	0	0	1	2
20	0	0	0	0	1	1
21	0	0	0	0	1	4
22	0	0	0	0	0	1
23	0	0	0	0	0	1
24	0	0	0	0	0	1
25	0	0	0	0	0	2
26	0	0	0	0	0	3
27	2	2	0	0	1	7
28	0	0	0	0	0	3
30	0	0	0	0	0	3
31	0	0	0	0	0	1
32	0	0	0	0	1	3
33	0	2	0	0	1	7
34	0	2	0	0	1	6
35	0	0	0	0	0	1
36	0	0	0	0	0	3
37	0	0	0	0	0	2
38	0	0	0	0	1	3
39	0	0	0	0	0	1
40	0	1	0	0	0	3
41	0	0	0	0	0	2
42	0	0	0	0	0	1
43	0	0	0	0	0	2
44	0	2	0	0	0	3
45	0	0	0	0	0	3
46	0	2	0	0	0	3
47	0	0	0	0	0	1
48	0	0	0	0	0	2
49	0	2	0	0	1	5

50	0	0	0	0	0	1
51	0	2	0	0	1	6
52	0	0	0	0	0	1
53	0	0	0	0	0	3
55	0	0	0	0	0	1

**video number**	**EQUUS-FAP**
1	control 5
2	patient 2 2 hours after NSAIDs Saturday
4	control 3
5	patient 4 second morning after surgery no NSAID
6	patient 7 day 1 after NSAIDs
7	control 4
8	control 6
10	control 22
11	patient 5 before NSAID
12	control 1
13	patient 8 before surgery
14	control 7
15	patient 2 after NSAIDs Sunday
16	patient 4 before surgery
17	control 8
18	patient 6 2 hours after surgery
19	control 2
20	control 9
21	patient 2 before NSAIDs Saturday
22	control 10
23	patient 9 after NSAIDs
24	control 11
25	patient 6 8 hours after surgery
26	control 19
27	patient 5 2 hours after NSAIDs
28	control 14
30	Patient 3
31	patient 7 day 2 before NSAIDs
32	control 15
33	patient 6 before surgery
34	control 18
35	patient 4 2 hours after surgery
36	control 21
37	patient 1 post NSAID
38	patient 9 before NSAIDs
39	control 12
40	patient 2 before NSAIDs Sunday
41	control 13
42	patient 7 after surgery before NSAIDs
43	patient 4 marning after surgery after NSAID
44	control 20
45	patient 6 morning after surgery
46	patient 8 2 hours after surgery

47 control 16
48 patient 14 morning after surgery before NSAIDs
49 patient 1 pre NSAID
50 patient 10 pre-euthanasia
51 controle 17
