# Supplementary material for: Objective Assessment of Acute Pain in Foals Using a Facial Expression-Based Pain Scale
Source: Animals (Basel). 2020 Sep 10;10(9):1610. doi: 10.3390/ani10091610 (PMC7552134; doi:10.3390/ani10091610)
Supplement: Supplementary file 1 [file animals-10-01610-s001.zip › supplementary material 1 animal details.pdf]

neonatal patients		condition	gender	age (days)	breed
	patient1	orthopaedic surgery	filly	14	Friesian
	patient2	orthopaedic surgery	colt	14	Thoroughbred
	patient3	bladder rupture	colt	3	Thoroughbred
	patient4	inguinal surgery post surgery	colt	10	Warmblood
	patient5	orthopaedic surgery	colt	14	Thoroughbred
	patient6	inguinal surgery post surgery	filly	12	Warmblood
	patient7	orthopaedic surgery	filly	14	Thoroughbred
	patient8	gastric ulcera	colt	14	Warmblood
	patient9	laminitis	filly	6	Warmblood
	patient10	septic knee joint	colt	5	pony
			mean	10,60	
			stdv	4,35	

neonatal healthy control foals					
	controle 1		colt	3	Warmblood
	çontrole 2		filly	5	Coldblood
	controle 3		filly	7	Warmblood
	controle 4		filly	3	Coldblood
	controle 5		filly	4	Warmblood
	controle6		filly	3	Warmblood
	controle 7		colt	5	Warmblood
	controle 8		colt	5	Warmblood
	controle 9		colt	7	Warmblood
	controle 10		filly	4	Warmblood
	controle 11		colt	6	haflinger
	controle 12		colt	5	Coldblood
	controle 13		filly	7	Warmblood
	controle 14		filly	14	Thoroughbred
	controle 15		colt	5	Coldblood
	controle 16		filly	4	Warmblood
	controle 17		filly	5	Warmblood
			mean	5,41	
			stdv	2,58	

**older patients**		**condition**	**gender**	**age (days)**	**breed**
	patient1	post colic SI volvulus	filly	28	Thoroughbred
	patient2	trauma	filly	112	Warmblood
	patient3	dental disorder post-surgery	filly	112	Warmblood
	patient4	umbilical hernia post surgery	filly	140	Warmblood
	patient5	phalanx 3 fracture	filly	98	Warmblood
	patient6	trauma	colt	140	Warmblood
	patient7	trauma	filly	140	Warmblood
	patient8	trauma	filly	126	Warmblood
	patient9	desmotomy check ligament post	filly	84	Warmblood
	patient10	abdominal rupture pre euthanas	filly	49	Thoroughbred
			mean	102,9	
			stdv	39,05	

**older healthy control foals**		**gender**	**age (days)**	**breed**
	controle 1	colt	42	Thoroughbred
	çontrole 2	colt	73	Warmblood
	controle 3	colt	73	Warmblood
	controle 4	colt	130	Warmblood
	controle 5	colt	100	Warmblood
	controle6	colt	157	Warmblood
	controle 7	colt	95	Warmblood
	controle 8	colt	91	Warmblood
	controle 9	colt	175	Warmblood
	controle 10	filly	114	Warmblood
	controle 11	filly	137	Warmblood
	controle 12	filly	73	Warmblood
	controle 13	filly	67	Warmblood
	controle 14	filly	65	Warmblood
	controle 15	filly	170	Warmblood
	controle 16	filly	122	Warmblood
	controle 17	filly	91	Warmblood
	controle 18	filly	120	Warmblood
	controle 19	filly	176	Warmblood
	controle 20	filly	115	Warmblood
	controle 21	filly	172	Warmblood
	controle 22	filly	111	Warmblood
		mean	112,23	
		stdv	39,76	
