# Supplementary material for: Objective Assessment of Acute Pain in Foals Using a Facial Expression-Based Pain Scale
Source: Animals (Basel). 2020 Sep 10;10(9):1610. doi: 10.3390/ani10091610 (PMC7552134; doi:10.3390/ani10091610)
Supplement: Supplementary file 1 [file animals-10-01610-s001.zip › supplementary material 2 pain scores neonatal foals Observer 1.pdf]

video number	head	eyelids	focus	nostrils	corners mouth/li	muscle tone head
1	0	0	0	1	0	0
2	0	0	0	1	1	0
3	0	0	0	0	0	0
4	0	0	0	1	0	0
5	0	0	0	0	1	0
6	0	0	0	1	0	0
7	0	0	0	0	2	0
8	0	0	0	0	0	0
9	0	0	0	0	2	0
10	0	0	0	0	0	0
11	0	0	0	1	0	0
12	0	0	0	0	2	0
13	0	0	0	1	1	0
14	1	1	1	1	2	2
15	0	0	0	0	0	0
16	1	1	1	0	0	0
17	0	0	0	1	0	0
18	0	0	0	1	1	0
19	0	0	0	0	0	0
20	1	0	1	0	2	1
21	0	0	0	0	0	0
22	1	1	1	0	2	0
23	0	0	0	1	0	0
24	0	0	0	0	0	0
25	0	0	1	0	1	2
26	0	0	1	0	0	0
27	0	0	0	0	0	1
28	0	0	0	0	0	0
29	0	0	0	0	0	0
30	0	0	0	0	0	0
31	0	1	1	0	0	0
32	1	1	1	0	1	0
33	0	0	0	0	1	0
34	0	0	0	1	0	0
35	1	1	1	0	2	1
36	0	0	0	0	0	0
37	0	0	0	0	0	0
38	0	0	0	0	1	0
39	0	1	1	0	0	0
40	0	0	0	0	0	0
41	0	0	0	0	0	0
42	1	1	1	0	1	0
43	0	0	0	0	0	0
44	0	0	0	0	0	0
45	0	0	0	1	0	0
46	0	0	0	0	1	0
47	0	0	0	1	0	0
48	0	0	0	0	2	0
49	0	0	0	1	0	0

50	0	0	0	0	0	0
51	0	0	0	1	1	0
52	0	0	0	0	0	0
53	1	1	1	1	2	2
54	0	1	1	0	0	0
55	0	0	0	0	0	0
56	1	0	1	0	2	2
57	0	0	0	1	0	0
58	0	0	0	0	0	0
59	0	0	0	1	0	0
60	0	0	0	0	0	0
61	0	0	0	0	0	0
62	0	1	1	0	0	0
63	0	0	0	0	2	2
64	0	0	0	0	0	0

**video number**	**yawning**	**lip smacking**	**teeth grinding**	**moaning**	**ears**	**total EQUUS-FAP score**
1	0	2	0	0	0	3
2	0	0	0	0	1	3
3	0	2	0	0	0	2
4	0	2	0	0	0	3
5	0	2	0	0	0	3
6	0	2	2	0	0	5
7	0	0	0	0	0	2
8	0	0	0	0	0	0
9	0	0	0	0	1	3
10	0	2	0	0	0	2
11	0	0	0	0	0	1
12	2	2	0	0	0	6
13	0	0	0	0	0	2
14	0	2	0	0	1	11
15	0	2	0	0	0	2
16	0	2	0	0	1	6
17	0	0	0	0	0	1
18	0	2	0	0	0	4
19	0	0	0	0	0	0
20	0	2	0	0	1	8
21	0	0	0	0	0	0
22	0	0	0	0	1	6
23	0	0	0	0	0	1
24	0	0	0	0	0	0
25	0	0	0	0	1	5
26	0	2	0	0	1	4
27	2	2	0	0	0	5
28	0	0	0	0	0	0
29	0	0	0	0	0	0
30	0	2	0	0	0	2
31	2	2	0	0	1	7
32	0	0	0	0	1	5

33	0	2	0	0	0	3
34	0	2	0	0	0	3
35	0	0	0	0	1	7
36	0	2	0	0	0	2
37	0	0	0	0	0	0
38	0	0	0	0	0	1
39	0	2	0	0	1	5
40	0	2	0	0	0	2
41	0	0	0	0	0	0
42	0	0	0	0	1	5
43	0	2	0	0	0	2
44	2	2	0	0	0	4
45	0	2	2	0	0	5
46	2	2	0	0	0	5
47	0	2	0	0	0	3
48	0	2	0	0	1	5
49	0	0	0	0	0	1
50	0	0	0	0	0	0
51	0	0	0	0	1	3
52	0	0	0	0	0	0
53	0	2	0	0	1	11
54	2	2	0	0	1	7
55	0	0	0	0	0	0
56	0	2	0	0	1	9
57	0	0	0	0	0	1
58	0	0	0	0	0	0
59	0	2	0	0	1	4
60	0	0	0	0	0	0
61	0	2	0	0	0	2
62	0	2	0	0	1	5
63	0	0	0	0	1	5
64	0	2	0	0	0	2

video number patients

2	patient 1 (before NSAID's)
5	patient 2 (before surgery)
7	patient 3 (after surgery, post NSAIDs)
9	patient 4 (after surgery, post NSAIDs)
11	patient 5 (after surgery, pre NSAIDs)
12	patient 3 (after OK, pre NSAIDs)
14	patient 6 (after surgery, pre NSAIDs)
16	patient 7 8 hours post NSAIDs
18	patient 1 (2,5 h after NSAIDs)
20	patient 8
22	patient 4 (after surgery, before NSAIDs)
25	patient 9 (9 h after NSAIDs)
27	patient 4 (before surgery)
30	patient 4 (before surgery)
32	patient 6 (after surgery, 1 hour after NSAIDs)

33	patient 3 (after surgery, post NSAIDs)
35	patient 4 (after surgery, before NSAIDs)
37	patient 5 (after surgery, pre NSAIDs)
39	patient 7 8 hours post NSAIDs
42	patient 6 (after surgery, 1 hour after NSAIDs)
44	patient 6 (after surgery, 1 hour after NSAIDs)
46	patient 3 (after OK, pre NSAIDs)
48	patient 4 (after surgery, post NSAIDs)
51	patient 1 (before NSAIDs)
53	patient 6 (after surgery, pre NSAIDs)
56	patient 8
59	patient 1 (2,5 h after NSAIDs)
61	patient 2 (before surgery)
63	patient 9 (9 h after NSAIDs)
64	patient 4 (before surgery)

**video number**	**controls**
1	foal 6
3	Foal 8
4	Foal 13
6	Foal 4
8	foal 16
10	foal 9
13	Foal 15
15	Foal 10
17	Foal 2
19	Foal 3
21	Foal 12
23	foal 17
24	foal 5
26	Foal 7
28	Foal 14
29	Foal 1
31	Foal 11
47	foal 6
40	Foal 8
34	Foal 13
45	Foal 4
52	foal 16
36	foal 9
57	Foal 15
43	Foal 10
49	Foal 2
41	Foal 3
50	Foal 12
38	foal 17
55	foal 5
62	Foal 7
58	Foal 14

60	Foal 1
54	Foal 11
