# Supplementary material for: Objective Assessment of Acute Pain in Foals Using a Facial Expression-Based Pain Scale
Source: Animals (Basel). 2020 Sep 10;10(9):1610. doi: 10.3390/ani10091610 (PMC7552134; doi:10.3390/ani10091610)
Supplement: Supplementary file 1 [file animals-10-01610-s001.zip › supplementary material 4 pain scores neonatal foals Observer 3.pdf]

video number	head	eyelids	focus	nostrils	corners mouth/li	muscle tone head
1	0	1	0	0	0	0
2	0	0	0	1	1	0
3	0	0	0	0	0	0
4	0	0	0	1	0	0
5	0	1	0	0	0	0
6	0	0	0	1	0	0
7	1	0	0	0	1	0
8	0	0	0	0	0	0
9	1	0	0	1	1	0
10	0	0	0	1	0	0
11	0	1	0	1	0	0
12	0	1	0	1	1	0
13	0	0	0	1	0	0
14	1	2	0	1	1	0
15	0	0	0	0	0	0
16	0	1	1	0	0	0
17	0	0	0	1	0	0
18	0	0	0	1	0	0
19	0	0	0	0	0	0
20	1	1	1	0	1	0
21	0	0	0	0	0	0
22	1	1	1	0	1	0
23	0	1	0	1	0	0
24	0	0	0	0	0	0
25	1	1	0	1	1	0
26	0	1	0	0	0	0
27	1	1	0	1	1	0
28	0	0	0	0	1	0
29	0	0	0	0	0	0
30	0	0	0	1	0	0
31	0	1	0	1	0	0
32	1	1	1	0	0	0

video number	yawning	lip smacking	teeth grinding	moaning	ears	total EQUUS-FAP score
1	0	2	0	0	0	3
2	0	0	0	0	2	4
3	0	0	0	0	1	1
4	0	2	0	0	0	3
5	0	2	0	0	0	3
6	0	2	0	0	0	3
7	0	0	0	0	0	2
8	0	0	0	0	0	0
9	0	0	0	0	1	4
10	2	0	0	0	1	4
11	0	0	0	0	0	2
12	2	2	0	0	0	7
13	0	0	0	0	0	1
14	0	2	0	0	2	9

15	0	0	0	0	0	0
16	0	2	0	0	1	5
17	0	0	0	0	0	1
18	0	2	0	0	1	4
19	0	0	0	0	1	1
20	0	2	0	0	1	7
21	0	0	0	0	0	0
22	0	0	0	0	1	5
23	0	0	0	0	0	2
24	0	0	0	0	0	0
25	0	0	0	0	1	5
26	0	2	0	0	1	4
27	2	2	0	0	0	8
28	0	0	0	0	0	1
29	0	0	0	0	0	0
30	0	2	0	0	0	3
31	2	2	0	0	1	7
32	0	0	0	0	2	5

**video number**	**patients**
2	patient 1 (before NSAID's)
5	patient 2 (before surgery)
7	patient 3 (after surgery, post NSAIDs)
9	patient 4 (after surgery, post NSAIDs)
11	patient 5 (after surgery, pre NSAIDs)
12	patient 3 (after OK, pre NSAIDs)
14	patient 6 (after surgery, pre NSAIDs)
16	patient 7 8 hours post NSAIDs
18	patient 1 (2,5 h after NSAIDs)
20	patient 8
22	patient 4 (after surgery, before NSAIDs)
25	patient 9 (9 h after NSAIDs)
27	patient 4 (before surgery)
30	patient 4 (before surgery)
32	patient 6 (after surgery, 1 hour after NSAIDs)
33	patient 3 (after surgery, post NSAIDs)
35	patient 4 (after surgery, before NSAIDs)
37	patient 5 (after surgery, pre NSAIDs)
39	patient 7 8 hours post NSAIDs
42	patient 6 (after surgery, 1 hour after NSAIDs)
44	patient 6 (after surgery, 1 hour after NSAIDs)
46	patient 3 (after OK, pre NSAIDs)
48	patient 4 (after surgery, post NSAIDs)
51	patient 1 (before NSAIDs)
53	patient 6 (after surgery, pre NSAIDs)
56	patient 8
59	patient 1 (2,5 h after NSAIDs)
61	patient 2 (before surgery)

63	patient 9 (9 h after NSAIDs)
64	patient 4 (before surgery)

video number controls

1	foal 6
3	Foal 8
4	Foal 13
6	Foal 4
8	foal 16
10	foal 9
13	Foal 15
15	Foal 10
17	Foal 2
19	Foal 3
21	Foal 12
23	foal 17
24	foal 5
26	Foal 7
28	Foal 14
29	Foal 1
31	Foal 11
47	foal 6
40	Foal 8
34	Foal 13
45	Foal 4
52	foal 16
36	foal 9
57	Foal 15
43	Foal 10
49	Foal 2
41	Foal 3
50	Foal 12
38	foal 17
55	foal 5
62	Foal 7
58	Foal 14
60	Foal 1
54	Foal 11
