# Supplementary material for: Objective Assessment of Acute Pain in Foals Using a Facial Expression-Based Pain Scale
Source: Animals (Basel). 2020 Sep 10;10(9):1610. doi: 10.3390/ani10091610 (PMC7552134; doi:10.3390/ani10091610)
Supplement: Supplementary file 1 [file animals-10-01610-s001.zip › supplementary material 5 pain scores older foals Observer 1.pdf]

video number	head	eyelids	focus	nostrils	corners mouth/li	muscle tone head
1	0	0	0	1	0	0
2	0	0	0	1	0	0
4	0	0	0	0	0	0
5	0	0	0	0	0	0
6	0	0	0	0	0	0
7	0	0	0	0	1	0
8	0	0	0	0	0	0
10	0	0	0	0	0	0
11	0	0	0	0	0	0
12	0	0	0	1	0	0
13	0	0	0	0	0	0
14	0	0	0	1	0	0
15	0	0	0	0	0	0
16	0	0	0	1	0	1
17	0	1	0	0	0	0
18	0	0	0	0	0	0
19	0	0	0	0	0	0
20	0	0	0	0	0	0
21	0	0	0	1	0	0
22	0	0	0	0	0	0
23	0	0	0	1	0	0
24	0	0	0	0	0	0
25	0	1	0	0	0	0
26	0	0	0	1	0	0
27	0	0	0	0	0	0
28	0	0	0	0	0	0
30	1	1	0	0	0	1
31	0	0	0	0	1	0
32	0	0	0	0	0	0
33	0	0	0	0	0	0
34	0	0	0	0	0	0
35	0	0	0	0	0	0
36	0	0	0	0	0	0
37	0	0	0	0	1	0
38	0	0	0	0	0	1
39	0	0	0	0	0	0
40	0	0	0	1	0	0
41	0	0	0	0	0	0
42	0	0	0	1	0	0
43	0	0	0	0	0	0
44	0	0	0	0	0	0
45	0	0	1	0	0	0
46	0	1	0	1	0	1
47	0	0	0	0	0	0
48	0	0	0	0	0	0
49	0	1	0	1	0	1
50	2	0	1	1	0	0
51	0	0	0	0	0	0
52	0	0	0	1	0	0

53	0	0	0	1	0	0
55	0	0	0	0	0	0
56	0	0	0	0	0	0
57	0	0	0	0	0	0
58	0	0	0	0	1	0
59	0	0	0	0	0	0
61	0	0	0	1	0	0
62	0	0	0	0	0	0
63	0	0	0	1	0	0
64	0	0	0	0	0	0
65	0	0	0	0	0	0
66	1	0	0	1	0	0
67	0	0	0	1	0	0
68	0	1	0	0	0	0
69	0	0	0	0	0	0
70	1	0	0	0	0	0
71	0	0	0	0	0	0
72	0	0	0	1	0	0
73	0	0	0	0	0	0
74	0	0	0	1	0	1
75	0	0	0	1	0	0
76	0	1	0	0	0	0
77	0	0	0	1	0	0
78	0	0	0	0	0	0
79	0	0	0	0	0	0
81	0	1	1	0	0	1
82	0	0	0	0	1	0
83	0	0	0	0	0	0
84	0	0	0	0	0	0
85	0	0	0	0	0	0
86	0	0	0	1	0	0
87	0	0	0	0	0	0
88	0	0	0	0	0	0
89	0	0	0	1	0	1
90	0	0	0	1	0	0
91	0	0	0	1	0	0
92	0	0	0	0	0	0
93	0	0	0	0	1	0
94	0	0	0	0	0	0
95	0	0	0	0	0	0
96	0	1	1	0	0	0
97	0	1	0	1	0	0
98	0	0	0	0	0	0
99	0	0	0	0	0	0
100	1	1	0	1	0	1
101	2	0	1	1	0	0
102	0	0	0	0	0	0
3	0	1	0	0	0	0
9	0	0	0	0	0	0

29	0	0	0	0	0	0
54	0	1	0	0	0	0
60	0	0	0	0	0	0
80	0	0	0	0	0	0

video number	yawning	lip smacking	teeth grinding	moaning	ears	total EQUUS-FAP score
1	0	0	0	0	0	1
2	0	0	0	0	0	1
4	0	0	0	0	0	0
5	0	2	0	0	0	2
6	0	2	0	0	1	3
7	0	0	0	0	0	1
8	0	2	0	0	0	2
10	0	0	0	0	0	0
11	0	0	0	0	0	0
12	0	2	0	0	0	3
13	0	0	0	0	1	1
14	0	0	0	0	0	1
15	0	0	0	0	1	1
16	0	0	2	0	0	4
17	0	0	0	0	1	2
18	0	0	0	0	1	1
19	0	0	0	0	1	1
20	0	0	0	0	0	0
21	0	0	0	0	0	1
22	0	0	0	0	0	0
23	0	0	0	0	0	1
24	0	0	0	0	0	0
25	2	0	0	0	2	5
26	0	0	0	0	0	1
27	0	2	0	0	0	2
28	0	0	0	0	0	0
30	0	2	0	0	1	6
31	0	2	0	0	1	4
32	0	0	0	0	0	0
33	0	0	0	0	0	0
34	0	2	0	0	0	2
35	0	0	0	0	1	1
36	0	2	0	0	0	2
37	0	2	0	0	1	4
38	0	2	0	0	0	3
39	0	0	0	0	0	0
40	0	2	0	0	0	3
41	0	2	0	0	0	2
42	0	0	0	0	0	1
43	0	0	2	0	0	2
44	0	0	0	0	0	0
45	0	0	0	0	1	2
46	0	2	0	0	1	6

47	0	0	0	0	0	0
48	0	2	0	0	1	3
49	0	0	0	0	0	3
50	0	0	0	0	0	4
51	0	0	0	0	0	0
52	0	0	0	0	0	1
53	0	0	0	0	0	1
55	0	0	0	0	0	0
56	0	2	0	0	0	2
57	0	2	0	0	1	3
58	0	0	0	0	0	1
59	0	2	0	0	0	2
61	0	0	0	0	1	2
62	0	0	0	0	0	0
63	0	2	0	0	1	4
64	0	0	0	0	1	1
65	0	0	0	0	0	0
66	0	0	0	0	1	3
67	0	2	0	0	0	3
68	0	0	0	0	1	2
69	0	0	0	0	1	1
70	0	0	0	0	1	2
71	0	0	0	0	0	0
72	0	0	0	0	0	1
73	0	0	0	0	0	0
74	0	0	0	0	1	3
75	0	0	0	0	0	1
76	2	0	0	0	2	5
77	0	0	0	0	0	1
78	0	2	0	0	0	2
79	0	0	0	0	0	0
81	0	2	0	0	1	6
82	0	2	0	0	1	4
83	0	0	0	0	0	0
84	0	0	0	0	0	0
85	0	0	0	0	1	1
86	0	0	0	0	0	1
87	0	2	0	0	0	2
88	0	2	0	0	1	3
89	0	0	0	0	1	3
90	0	0	0	0	0	1
91	0	2	0	0	0	3
92	0	2	0	0	0	2
93	0	0	0	0	1	2
94	0	0	2	0	0	2
95	0	0	0	0	0	0
96	0	0	0	0	1	3
97	0	2	0	0	1	5
98	0	0	0	0	0	0
99	0	2	0	0	1	3

100	0	0	0	0	1	5
101	0	0	0	0	0	4
102	0	0	0	0	0	0
3	0	2	0	0	2	5
9	0	2	0	0	0	2
29	0	2	0	0	2	4
54	0	2	0	0	2	5
60	0	2	0	0	1	3
80	0	2	0	0	2	4

**video number**	**EQUUS-FAP**
1	control 5
2	patient 2 2 hours after NSAIDs Saturday
4	control 3
5	patient 4 second morning after surgery no NSAID
6	patient 7 day 1 after NSAIDs
7	control 4
8	control 6
10	control 22
11	patient 5 before NSAID
12	control 1
13	patient 8 before surgery
14	control 7
15	patient 2 after NSAIDs Sunday
16	patient 4 before surgery
17	control 8
18	patient 6 2 hours after surgery
19	control 2
20	control 9
21	patient 2 before NSAIDs Saturday
22	control 10
23	patient 9 after NSAIDs
24	control 11
25	patient 6 8 hours after surgery
26	control 19
27	patient 5 2 hours after NSAIDs
28	control 14
30	Patient 3
31	patient 7 day 2 before NSAIDs
32	control 15
33	patient 6 before surgery
34	control 18
35	patient 4 2 hours after surgery
36	control 21
37	patient 1 post NSAID
38	patient 9 before NSAIDs
39	control 12
40	patient 2 before NSAIDs Sunday

41 control 13
42 patient 7 after surgery before NSAIDs
43 patient 4 marning after surgery after NSAID
44 control 20
45 patient 6 morning after surgery
46 patient 8 2 hours after surgery
47 control 16
48 patient 14 morning after surgery before NSAIDs
49 patient 1 pre NSAID
50 patient 10 pre-euthanasia
51 controle 17

52 control 5
53 patient 2 2 hours after NSAIDs saturday
55 control 3
56 patient 4 second morning after surgery no NSAID
57 patient 7 first day after surgery before NSAIDs
58 control 4
59 control 6
61 control 22
62 patient 5 before NSAIDs
63 control 1
64 patient 8 before surgery
65 control 7
66 patient 2 after NSAIDs sunday
67 patient 4 before surgery
68 control 8
69 patient 6 2 hours after surgery
70 çontrol 2
71 control 9
72 patient 2 before NSAID Saturday
73 control 10
74 patient 9 after NSAIDs
75 control 11
76 patient 6 8 hours after surgery
77 control 19
78 patient 5 2 hours after NSAIDs
79 control 14
81 Patient 3
82 patient 7 day 2 before NSAIDs
83 control 15
84 patient 6 before surgery
85 control 18
86 patient 4 2 hours after surgery
87 control21
88 patient 1 after NSAID
89 patient 9 before NSAIDs
90 control 12
91 patient 2 before NSAIDs sunday
92 control 13

93 patient 7 2 hours after surgery before NSAIDs
94 patient 4 morning after surgery after NSAIDs
95 control 20
96 patient 6 first morning after surgery
97 patient 8 2 hours after surgery
98 control 16
99 patient 14 morning after surgery before sNSAIDs
100 patient 1 before NSAID
101 patient 10 pre-euthanasia
102 control 17

3 neonatal after surgery before NSAID
9 neonatal 24 hours after surgery before NSAIDs
29 neonatal 24 hours after surgery after NSAIDs

54 neonatal after surgery before NSAID
60 neonatal 24 hours after surgery before NSAIDs
80 neonatal 24 hours after surgery after NSAIDs
